# Intermittent protein restriction before but not after the onset of diabetic kidney disease attenuates disease progression in mice

**DOI:** 10.3389/fnut.2024.1383658

**Published:** 2024-06-26

**Authors:** Xiaoyue Peng, Min Liu, Yijie Wu, Wenying Fan, Yi Hou, Yan Kong, Yajin Liu, Xuejiao Zhang, Chunyan Shan, Haipeng Sun, Yanhui Yang

**Affiliations:** ^1^NHC Key Laboratory of Hormones and Development, Tianjin Key Laboratory of Metabolic Diseases, Chu Hsien-I Memorial Hospital and Tianjin Institute of Endocrinology, Tianjin Medical University, Tianjin, China; ^2^Center for Cardiovascular Diseases, The Province and Ministry Co-Sponsored Collaborative Innovation Center for Medical Epigenetics, Tianjin Medical University, Tianjin, China

**Keywords:** diabetic kidney disease, intermittent protein restriction, low protein diet, early intervention, cell-cell junction

## Abstract

**Background:**

High dietary protein intake exacerbates proteinuria in individuals with diabetic kidney disease (DKD). However, studies on the impacts of low protein diet (LPD) on DKD have yielded conflicting results. Furthermore, patient compliance to continuous protein restriction is challenging.

**Objective:**

The current study aims to investigate the effects of intermittent protein restriction (IPR) on disease progression of DKD.

**Methods:**

Diabetic KK-Ay mice were used in this study. For the IPR treatment, three consecutive days of LPD were followed by four consecutive days of normal protein diet (NPD) within each week. For early intervention, mice received IPR before DKD onset. For late intervention, mice received IPR after DKD onset. In both experiments, age-matched mice fed continuous NPD served as the control group. Kidney morphology, structure and function of mice in different groups were examined.

**Results:**

Intermittent protein restriction before DKD onset ameliorated pathological changes in kidney, including nephromegaly, glomerular hyperfiltration, tubular injuries and proteinuria, without improving glycemic control. Meanwhile, IPR initiated after DKD onset showed no renoprotective effects despite improved glucose homeostasis.

**Conclusion:**

Intermittent protein restriction before rather than after DKD onset protects kidneys, and the impacts of IPR on the kidneys are independent of glycemic control. IPR shows promise as an effective strategy for managing DKD and improving patient compliance.

## 1 Introduction

Diabetic kidney disease (DKD), which progresses from an initially elevated glomerular filtration rate (GFR) to albuminuria and reduced GFR, is the leading cause of end-stage renal disease (ESRD) and the strongest predictor of mortality in diabetic patients (1–4). It is estimated that 783 million adults worldwide will have diabetes by 2045. With up to 40% of individuals with type 2 diabetes developing DKD, the incidence of DKD will continue to be an unmet clinical need (5, 6).

High protein intake is a well-established risk factor for the progression of DKD. In DKD, high protein intake can lead to significant increases in renal blood flow and GFR, which are thought to precede the loss of nephron units (7–12). Such increases can be prevented by protein restriction (PR) (13–16). In individuals with DKD, low protein intake may slow the progression of DKD, delay the initiation of renal replacement therapy and reduce urinary albumin excretion (17–28). It has been suggested that people with stage 3 to 5 of DKD should aim for a reduced dietary protein intake (DPI) of 0.6 to 0.8 g/kg body weight (BW) per day (29, 30).

Despite the potential benefits, patient compliance with continuous PR limits its application (19). There was an inverse relationship between patient compliance and urinary albumin excretion (28). More than a third of individuals do not have good compliance with continuous PR (20, 24). This brings intermittent protein restriction (IPR) intervention into consideration. An intermittent protein-restricted, calorie-restricted diet, known as the fasting mimicking diet (FMD), improved patient compliance and reduced dietary fatigue (31, 32). IPR has been shown to benefit insulin sensitivity similarly to continuous PR (33). It remains unclear whether IPR can provide beneficial effects on the progression of DKD.

Early intervention of DKD is crucial for improving renal outcomes. The rate of progression to ESRD in diabetic individuals with macroalbuminuria is about 14 times faster than that in those with other kidney diseases. The risk of all-cause mortality increases significantly as DKD progresses (34). Medication initiated after stage 3 or 4 of DKD may not slow disease progression as effectively as that started at the onset of microalbuminuria (35–37). Furthermore, in diabetic individuals, PR yields better results when implemented in the early stages of kidney disease or even before its onset (38, 39). Therefore, early detection and intervention is urgently needed to delay the onset and progression of DKD.

In this study, we used the KK-Ay mice, which closely mimic type 2 diabetic kidney disease, to investigate the effects of IPR on renal morphology, structure and function in DKD. The results showed that IPR before but not after the onset of DKD attenuated DKD progression.

## 2 Materials and methods

### 2.1 Mice

The study was conducted according to the guidelines of the Declaration of Helsinki, and approved by the Committee on Ethics in the Care and Use of Laboratory Animals of Chu Hsien-I Memorial Hospital, Tianjin Medical University (Approval No. DXBYY-IACUC-2022070, approved on 1 March 2022). Nine-week-old male KK-Ay mice, which spontaneously develop diabetes, were obtained from HFK Bioscience Co. Ltd., Beijing. The animals were maintained in specific-pathogen-free, temperature-controlled (23 ± 1°C) facilities with optimal humidity, and a 12-h light/dark cycle at the Tianjin Key Laboratory of Metabolic Diseases, Chu Hsien-I Memorial Hospital and Tianjin Institute of Endocrinology, Tianjin Medical University.

### 2.2 Mouse diet and intermittent protein restriction (IPR)

The normal protein diet (NPD) and the specialized low protein diet (LPD) based on the NPD were obtained from HFK Bioscience Co. Ltd., Beijing (Table 1). The NPD provided 16.46% of calories from protein, 37.89% of calories from carbohydrates, and 45.65% of calories from fat. The LPD provided 5% of calories from protein. The reduced calories from protein in LPD were replaced by calories from carbohydrates, while calories from fat were held fixed, making the two diets isocaloric (4.25 kcal/g). In the IPR group, mice were fed LPD for three consecutive days followed by NPD for four consecutive days in each week, as previously described (33). For early intervention, mice were randomized to receive immediate intervention before the onset of DKD. For late intervention, mice received intervention after the onset of DKD when their urinary albumin to creatinine ratio (ACR) exceeded 300 mg/g in the absence of elevated serum creatinine levels. In both experiments, age-matched mice fed continuous NPD served as controls. All mice had *ad libitum* access to water and food except as indicated in the procedures below.

**TABLE 1:** Comparing the nutritional and caloric composition of two diets.

	Normal protein diet	Low protein diet
Protein (%)	16.46	5
Carbohydrate (%)	37.89	49.35
Fat (%)	45.65	45.65
Energy value (kcal/g)	4.25	4.25

### 2.3 Measurement of blood glucose and insulin

Mice were fasted for 6 h before blood glucose was measured. Plasma insulin levels were measured using a murine enzyme-linked immunosorbent assay (ELISA) kit from Jiangsu Meimian Industrial Co., Ltd., Jiangsu, China. The homeostasis model assessment of insulin resistance (HOMA-IR) was calculated.

### 2.4 Intraperitoneal glucose tolerance testing (IPGTT)

After overnight fasting, a single dose of 1 g/kg BW glucose was injected intraperitoneally and blood glucose levels were measured at various times. The area under the curve (AUC) is then developed to quantify the overall increase in blood glucose during the test.

### 2.5 Biochemical measurements

Immediately after anesthesia, whole blood was collected from the hearts of mice and placed in EDTA-K2-treated tubes. The blood was then centrifuged at 3,500 rpm for 10 min to separate the plasma supernatant, which was stored at −80°C for further experiments. The levels of plasma urea nitrogen, albumin, triglycerides (TG), total cholesterol (TC) and urinary retinol binding protein (RBP) were measured. Plasma levels of low-density lipoprotein cholesterol (LDL-C), creatinine and cystatin C were determined using murine ELISA kits (Jiangsu Meimian Industrial Co., Ltd.). The levels of urinary albumin, creatinine, Kim-1, NGAL, podocin and nephrin were also measured using ELISA kits from Jiangsu Meimian Industrial Co., Ltd.

### 2.6 Body composition analysis

Body composition analysis in mice was performed using quantitative magnetic resonance (QMR).

### 2.7 Glomerular filtration rate measurement

Mice were weighed and anesthetized with isoflurane. They were then injected retro-orbitally with FITC-labeled inulin (2 μL/g of BW). Blood samples were taken from the tail vein at various times. After centrifugation, the plasma supernatant was transferred to EP tubes and diluted with HEPES buffer (pH 7.4). The fluorescence intensity of the diluted plasma samples was measured using a microplate reader. Glomerular filtration rate (GFR) was calculated using GraphPad Prism software version 9.4.1.

### 2.8 Morphological analysis

After sacrifice, unilateral mouse kidney tissues were fixed in 4% paraformaldehyde and embedded in paraffin. Tissue sections were stained with hematoxylin and eosin (HE), Masson’s trichrome (MT) and periodic acid-Schiff (PAS). Tissue morphological changes were observed under an inverted microscope and photographed with the attached camera. Each staining was performed on 5 independent slides.

### 2.9 RNA-seq

High-throughput RNA sequencing (RNA-seq) method was used by LC Bio Technology CO., Ltd. (Hangzhou, China) to obtain gene expression profiles in our study. Total RNA was extracted from kidney tissues and subjected to mRNA enrichment, cDNA library construction and Illumina sequencing. The resulting reads were aligned to the reference genome and gene expression levels were quantified using Fragments Per Kilobase of exon model per Million mapped fragments (FPKM). Differential gene expression analysis was performed to identify significant changes. Pathway and functional enrichment analyses were performed using the Gene Ontology (GO) and Kyoto Encyclopedia of Genes and Genomes (KEGG) databases in R, with statistical significance determined using the hypergeometric test (*p*-value < 0.05).

### 2.10 Antibodies and immunoblotting

The antibody against E-cadherin was purchased from Proteintech (Wuhan, China). Antibodies to ZO-1 and occludin were purchased from Abmart (Abmart Shanghai Co., Ltd., Shanghai, China). The antibody against TGF-β was purchased from PTMab (Jingjie PTM BioLab Co., Ltd., Hangzhou, China). The immunoblotting protocol used in this study has been described previously (40). ImageJ software was used for image analysis.

### 2.11 Immunohistochemistry

For ZO-1 immunohistochemical staining, kidney sections were incubated in a humidified chamber with anti-ZO-1 antibody (catalog number ab221546, Abcam) and secondary antibody. The sections were then stained with a chromogenic substrate and hematoxylin (Beijing Solarbio Science & Technology Co., Ltd.). Multiple images (3 to 13) per section were captured using an Olympus BX51 microscope (Olympus Corporation, Tokyo, Japan).

### 2.12 Statistical analysis

All data are expressed as mean ± standard error of the mean (SEM). Statistical analysis was performed using an unpaired *t*-test where appropriate. Data that did not follow a normal distribution were tested by the nonparametric Mann–Whitney U test.

## 3 Results

### 3.1 IPR after the onset of DKD improved metabolic health

**FIGURE 1:**
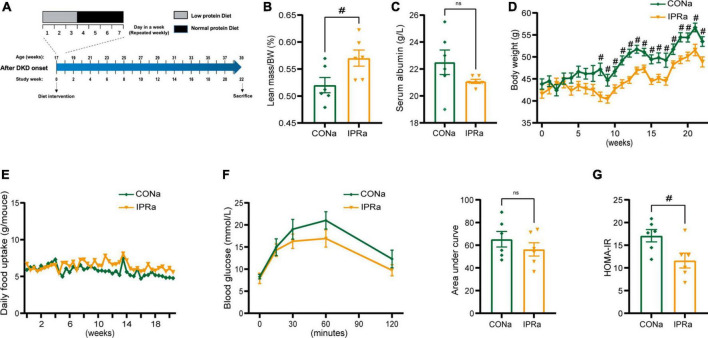
The beneficial metabolic effects of intermittent protein restriction (IPR) after the onset of diabetic kidney disease (DKD). **(A)** Protocol for investigating the influence of IPR after the onset of DKD on the renal outcomes in KK-Ay mice. **(B)** Lean mass adjusted by body weight (BW). **(C)** Serum albumin change after a 6-h fasting. **(D)** Changes of body weight during the experiment period. **(E)** Daily food consumption of every mouse during the experiment period. **(F)** Changes in blood glucose levels over time following intraperitoneal glucose injection. And area under the curve (AUC) of the intraperitoneal glucose tolerance test (IPGTT) was calculated. **(G)** Graphical representation of the homeostasis model assessment of insulin resistance (HOMA-IR) index. Data are represented as mean ± SEM (*n* = 6). ^#^*p* < 0.05, ns indicates a non-significant *p*-value. Two groups of mice were included: diabetic male KK-Ay mice receiving a continuous normal protein diet (CONa), and mice receiving IPR intervention (IPRa). IPRa versus CONa, by unpaired *t*-tests.

We first investigated the effects of IPR intervention after the onset of DKD (IPRa). Seventeen-week-old KK-Ay mice with elevated urinary albumin excretion above 300 mg/g creatinine without elevated serum creatinine levels (not shown) were treated with either continuous NPD or IPR for 22 weeks (Figure 1A). Relative lean body mass and serum albumin levels were not reduced after IPR intervention. These results suggested that IPR did not cause malnutrition in mice (Figures 1B, C). Interestingly, while calories from carbohydrates were used to replace calories from protein in LPD with a higher glycemic index, IPR initiated after the onset of DKD reduced body weight gain and improved glucose tolerance and insulin resistance (Figures 1D–G).

### 3.2 IPR after the onset of DKD did not improve renal outcomes

**FIGURE 2:**
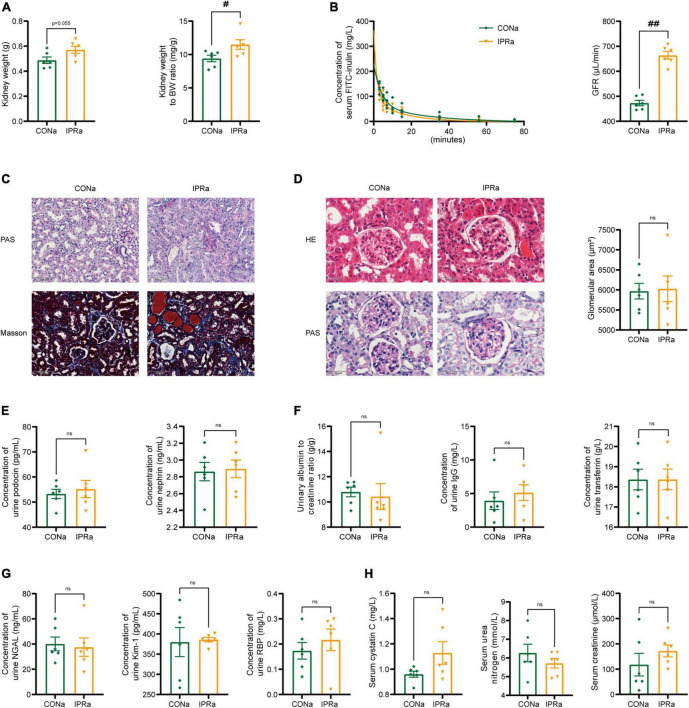
IPR after the onset of DKD did not improve renal outcomes. **(A)** Kidney weight and kidney index calculated as the kidney weight adjusted for BW after 22-week dietary intervention. **(B)** Concentrations of serum FITC-labeled inulin at various time points after retro-orbital injection. And glomerular filtration rate (GFR) was calculated based on the clearance of serum FITC-labeled inulin. **(C)** Representative images of PAS and MT staining in renal tubules (scale bar, 100 μm). **(D)** Representative images of H&E and PAS staining in glomeruli (scale bar, 50 μm), and quantitative analysis of glomerular size (25 glomeruli per mouse were analyzed, *n* = 6). **(E)** Urinary podocin and nephrin excretion levels. **(F)** Urinary albumin to creatinine ratio, immunoglobulin G (IgG) and transferrin (TF) excretion levels. **(G)** Levels of urinary neutrophil gelatinase-associated lipocalin (NGAL), kidney injury molecule-1 (Kim-1) and retinol binding protein (RBP) excretion. **(H)** Plasma cystatin C, urea nitrogen and creatinine levels after IPR intervention. Data are represented as mean ± SEM (*n* = 6). ^#^*p* < 0.05, ^##^*p* < 0.01, ns indicates a non-significant *p*-value. IPRa versus CONa.

Despite the beneficial metabolic effects, kidney weight, kidney index and GFR measured by serum FITC-labeled inulin concentrations were increased but not decreased in IPRa mice, compared with those in control group (Figures 2A, B). Mesangial matrix expansion and interstitial collagen deposition were more evident in IPRa mice than those in the control group (Figures 2C, D). Glomerular size was not significantly changed (Figure 2D). Podocin and nephrin are major protein components of the podocyte slit diaphragm, an important part of the glomerular filtration barrier (GFB). The urinary levels of podocin and nephrin were not reduced by IPR (Figure 2E). The excretion of urinary albumin, immunoglobulin G (IgG) and transferrin (TF) was not reduced following IPR (Figure 2F). Tubular injury markers including urinary neutrophil gelatinase-associated lipocalin (NGAL), kidney injury molecule-1 (Kim-1) and retinol binding protein (RBP) in the urine were not changed by IPR (Figure 2G). Serum cystatin C, urea nitrogen and creatinine levels were not decreased (Figure 2H). Together, these results showed that IPR starting after the onset of DKD failed to improve renal outcomes, regardless of improved glycemic control.

### 3.3 IPR before the onset of DKD improved renal outcomes

**FIGURE 3:**
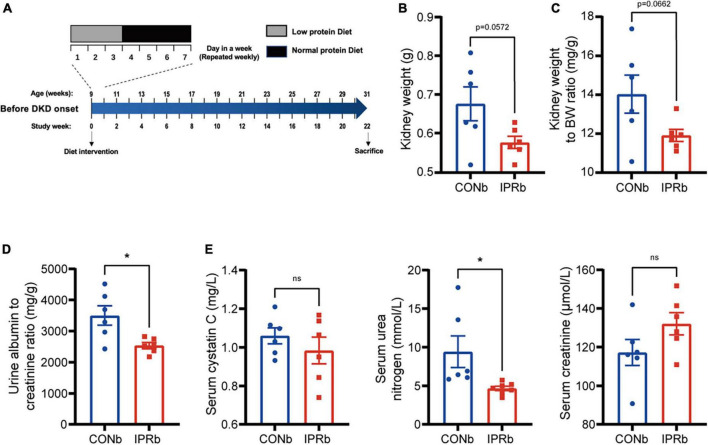
IPR before the onset of DKD improved renal outcomes. **(A)** Protocol for investigating the influence of IPR before the onset of DKD on the renal outcomes in KK-Ay mice. **(B)** Weight of kidneys. **(C)** Kidney index. **(D)** Urinary albumin to creatinine ratio after 22-week diet intervention. **(E)** Serum cystatin C, urea nitrogen and creatinine levels at the end of intervention. Data are represented as mean ± SEM (*n* = 6). *p < 0.05, ns indicates a non-significant *p*-value. Two groups of mice were included: mice fed a continuous normal protein diet (CONb), and mice fed IPR (IPRb) before DKD onset. IPRb versus CONb, by unpaired *t*-tests.

To determine whether IPR starting before the onset of DKD (IPRb) could slow DKD progression, KK-Ay mice at nine weeks of age that did not develop proteinuria were treated with either continuous NPD or IPR for 22 weeks (Figure 3A). In IPRb mice, kidney weight, kidney index and urinary albumin excretion (Figures 3B–D) were significantly reduced, compared with those in NPD-treated mice. Notably, despite the reduction in kidney volume, serum urea nitrogen and creatinine levels did not increase significantly (Figure 3E). Similarly, there was no difference in serum cystatin C levels between the IPRb group and the control group (Figure 3E). Together, these results demonstrated the protective effects of initiating IPR before the onset of early DKD.

### 3.4 IPR before the onset of DKD protected both glomeruli and tubules

**FIGURE 4:**
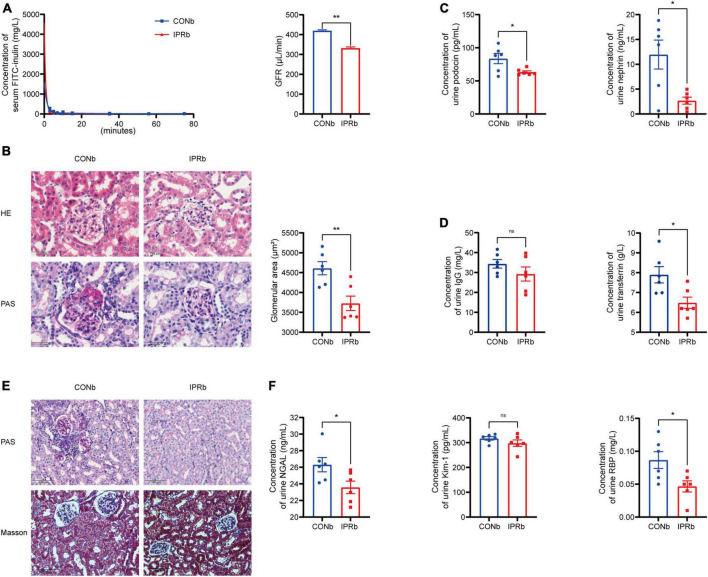
IPR before the onset of DKD protected both glomeruli and tubules. **(A)** Concentrations of FITC-labeled inulin at different time after retro-orbital injection. GFR was calculated. **(B)** Representative images of glomeruli stained with H&E and PAS (scale bar, 50 μm). Quantitative analysis of glomerular size was performed (25 glomeruli per mouse, *n* = 6). **(C)** Levels of urinary podocin and nephrin excretion. **(D)** Levels of urinary IgG and TF excretion. **(E)** Representative images of renal tubules stained with PAS and MT (scale bar, 100 μm). **(F)** Levels of urinary NGAL, Kim-1 and RBP excretion. Data are represented as mean ± SEM (*n* = 6). *p < 0.05, **p < 0.01, ns indicates a non-significant *p*-value. IPRb versus CONb.

Given that the impairment of glomerular filtration barrier, manifested by protein leakage from the bloodstream into the urine, is a strong risk factor for the progression of DKD to ESRD, we investigated the effects of IPRb on the glomeruli. The clearance of serum FITC-labeled inulin, the gold standard of kidney function, was used to measure GFR. In IPRb mice, measured GFR, glomerular volumes and mesangial matrix expansion were reduced compared with those in control mice (Figures 4A, B). Urinary podocin, nephrin, IgG, and TF levels were decreased by IPR started before the onset of DKD (Figures 4C, D). In the tubules, pathological changes such as infiltration of inflammatory cells and deposition of collagen fibers in the peritubular region were evident in the DKD mice (Figure 4E), which were attenuated by IPRb. The excretion of NGAL and RBP in the urine was reduced in IPRb group as well (Figure 4F). Together, these results highlighted the beneficial effects of IPRb in attenuating both glomerular and tubular damages.

### 3.5 IPR before the onset of DKD did not improve glycemic control

**FIGURE 5:**
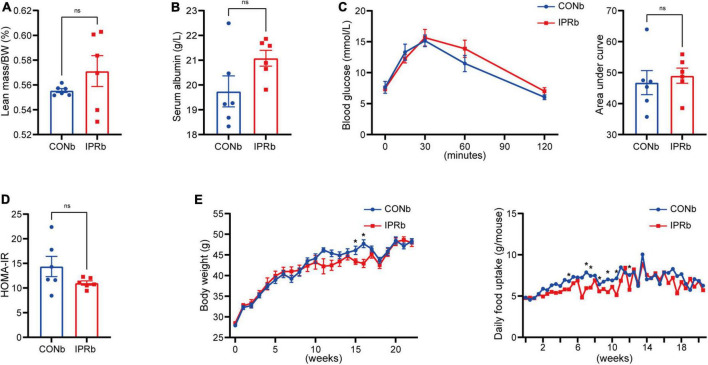
IPR before the onset of DKD did not improve glycemic control. **(A)** Lean mass adjusted by BW. **(B)** Serum albumin changes after a 6-h fasting. **(C)** Dynamic changes in blood glucose levels after intraperitoneal glucose injection and AUC of the IPGTT. **(D)** Graphical representation of the HOMA-IR index. **(E)** Body weight change and daily food consumption of every mouse throughout the study duration. Data are represented as mean ± SEM (*n* = 6). *p < 0.05, ns indicates a non-significant *p*-value, IPRb versus CONb.

Given the importance of glycemic control in slowing the progression of DKD, we tested whether the renoprotective effects of IPR before DKD onset resulted from glycemic control. IPR initiated before DKD onset did not result in decreased lean body mass or serum albumin levels (Figures 5A, B). Glucose tolerance and insulin resistance were not improved by IPRb (Figures 5C, D). Furthermore, IPR started before DKD onset did not reduce body weight gain or food intake (Figure 5E). Taken together, these findings suggested that the beneficial effects of IPRb on the kidneys were independent of glycemic control.

### 3.6 Cell-cell junction was improved before but not after DKD onset

**FIGURE 6:**
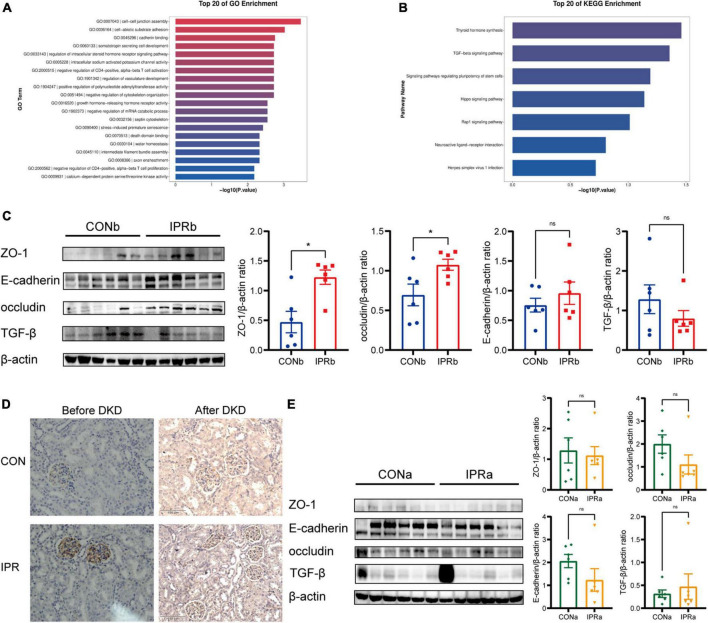
Cell-cell junction was improved by early IPR initiated before DKD onset. **(A)** GO analysis of differentially expressed genes in kidney tissues of which IPR started before DKD onset, focusing on biological processes. **(B)** KEGG analysis of pathways involved of kidney tissues. **(C)** Representative immunoblotting images illustrating the expression levels of ZO-1, E-cadherin, occludin, TGF-β and β-actin in kidney tissues of which IPR started before DKD onset (*n* = 6). Quantitative analysis of each protein expression level is presented, normalized to β-actin expression levels. **(D)** Immunostaining of ZO-1 in kidney tissues of which IPR started before or after DKD onset, with a scale bar representing 100 μm. **(E)** Representative immunoblotting images illustrating the expression levels of ZO-1, E-cadherin, occludin, TGF-β and β-actin in kidney tissues of which IPR started after DKD onset (*n* = 6). Quantitative analysis of each protein expression level is presented, normalized to β-actin expression levels. Data presented as mean ± SEM. Statistical significance indicated by ^#^*p* < 0.05, while ns indicates a non-significant *p*-value. IPRa versus CONa. *p < 0.05, ns indicates a non-significant *p*-value. IPRb versus CONb.

To investigate the potential mechanism underlying early IPR’s beneficial effects, we performed transcriptomic analysis of kidney tissues. Differentially expressed genes were particularly enriched in cell-cell junction assembly (GO: 0007043), cadherin binding (GO: 0045296), and adherens junction (GO: 0005912) (Figure 6A). Thyroid hormone synthesis (mmu04918) and TGF-beta signaling pathway (mmu04350) were identified as significantly differentially expressed pathways by KEGG pathway enrichment analysis. All genes mapped to these KEGG pathways were upregulated in the IPRb group, compared with those in the control group (Figure 6B). Selected results from the profile analyses were verified by Western blot, confirming the upregulation of ZO-1, occludin and E-cadherin, and the suppression of TGF-β (Figure 6C). ZO-1 upregulation was further supported by immunohistochemical staining (Figure 6D). On the other hand, IPR started after the onset of DKD did not alter the protein expression of ZO-1, occludin, E-cadherin, and TGF-β (Figure 6E). Immunohistochemical staining for ZO-1 in kidney tissues showed no significant differences. Together, these results suggested that early IPR intervention before DKD onset improved renal outcomes through protection of cell-cell junction.

## 4 Discussion

In the current study, we found that IPR before but not after DKD onset attenuated its progression. IPR before DKD onset ameliorated pathophysiological changes of DKD without improving glycemic control. On the other hand, IPR administered after the onset of DKD showed no significant renal protection despite the improved glucose homeostasis.

A study by Kitada et al. (41) showed improved kidney function when IPR was administered in advanced DKD. In contrast, the levels of serum creatinine and cystatin C were not significantly increased in mice when IPR started after DKD onset in the current study, but GFR was elevated, suggesting that different stages of DKD were involved. And in that study, the intervention was performed with an alternation between a high protein diet and LPD, different from the transition from NPD to LPD used in the current study. This diet difference may contribute to the different effects of IPR on renal outcomes.

Age-related protein absorption may play a role in the different effects of IPRa and IPRb on DKD. Despite the consensus that LPD benefits stages 3 to 5 DKD, our results showed that IPR started after DKD onset at the age of 17 weeks did not improve renal outcomes, in agreement with a study by Meloni et al. (39). IPR initiated at 9 weeks of age before the onset of DKD ameliorated the pathophysiological changes of DKD. This is consistent with a previous report suggesting that people under 65 years of age may benefit from LPD, whereas the older groups may not (42). Age-related malabsorption of proteins and amino acids can lead to malnutrition (43). The benefits of LPD may potentially be compromised by the adverse effects of malnutrition in older animals.

The lack of protection against DKD in the IPRa group could also be due to the short duration of the intervention. Studies have shown that the short-term effects of treatments, including PR, may differ from the long-term benefits on the progression of kidney disease (44). In the modification of diet in renal disease (MDRD) trial, long but not short LPD treatment showed a significant effect of PR in slowing GFR decline (45, 46). It is possible that extending the duration of IPRa intervention could show protective effects on DKD. Further research is needed to assess the long-term effects of IPRa.

The pathological changes of DKD at early or late stage may also attribute to the different effects of IPRa and IPRb. In the early stages of DKD, albuminuria is likely resulted from a local intrarenal hemodynamic effect rather than structural changes in the kidney. Early dietary intervention may reduce albuminuria and delay disease progression. In the late stages of DKD, the progressive structural damage becomes irreversible. Interventions at this advanced stage are less effective than early interventions (36, 47). This may partly explain the differences observed in the current study between early and late IPR intervention. Levels of albuminuria were significantly higher when IPRa was initiated than those when IPRb was initiated.

The amount of food taken by the control group and the IPR group showed no significant differences during LPD and NPD feeding periods, supporting lower total protein intake in the IPR-treated group. The reduced protein intake was further supported by the reduction in serum urea nitrogen levels in IPR group. Of note, there was no decrease in plasma albumin levels or lean mass after long-term IPR. These results suggested that the IPR approach effectively reduces protein intake while mitigating the risk of malnutrition associated with prolonged implementation of LPD (48).

IPR has been proposed as a potential strategy to improve glucose homeostasis (33), which is crucial for the treatment of DKD. Interestingly, our results showed that implementation of IPR before the onset of DKD slowed DKD progression without improving glycemic control. On the other hand, IPR after DKD onset showed no protective effect on DKD progression while improving glucose homeostasis. The improvement in glycemic control occurred despite the higher glycemic index of LPD. These results suggested that the effects of IPR on renal outcomes and glucose homeostasis were not correlated (49) and early IPR protected kidney through glycemia-independent mechanism.

Cell-cell junctions are important components for the integrity of GFB to prevent serum protein leakage into the urine (50). The upregulated expression levels of intracellular junction proteins ZO-1, occludin, and E-cadherin may partly contribute to the improved renal outcomes after IPR initiated before DKD onset. IPR initiated after DKD onset did not improve intracellular junctions, which may partly explain the lack of improvement of renal outcomes. The TGF-β signaling pathway is closely associated with intracellular junctions (51). The decrease in TGF-β expression levels was observed in the mice treated with IPR before the onset of DKD, suggesting that the TGF-β signaling pathway may be involved in the beneficial effects.

In conclusion, our research suggested that IPR held great promise as an effective strategy for the management of DKD independently of glycemic control. Importantly, early rather than late IPR intervention provided significant renal protection while mitigating the risk of malnutrition. The different renal outcomes of IPR intervention started at different time underscored the importance of bringing forward the timing of PR intervention to slow the progression of DKD.

## Data availability statement

The raw sequence data reported in this paper have been deposited in the NCBI Short Read Archive (SRA) with bioproject accession number (PRJNA1123475). The raw data supporting the conclusions of this article are available upon request from the corresponding author, without undue reservation.

## Ethics statement

The animal study was approved by the Committee on Ethics in the Care and Use of Laboratory Animals of Chu Hsien-I Memorial Hospital, Tianjin Medical University. The study was conducted in accordance with the local legislation and institutional requirements.

## Author contributions

XP: Formal analysis, Investigation, Writing – original draft. ML: Writing – review & editing. YW: Writing – review & editing. WF: Writing – review & editing. YH: Writing – review & editing. YK: Writing – review & editing. YL: Resources, Writing – review & editing. XZ: Writing – review & editing. CS: Conceptualization, Writing – review & editing. HS: Conceptualization, Writing – review & editing. YY: Conceptualization, Formal analysis, Investigation, Writing – review & editing.
